# Dog Owners' Interaction Styles: Their Components and Associations with Reactions of Pet Dogs to a Social Threat

**DOI:** 10.3389/fpsyg.2016.01979

**Published:** 2016-12-20

**Authors:** Giulia Cimarelli, Borbála Turcsán, Zsófia Bánlaki, Friederike Range, Zsófia Virányi

**Affiliations:** ^1^Clever Dog Lab, Comparative Cognition, Messerli Research Institute, University of Veterinary Medicine of ViennaVienna, Austria; ^2^Wolf Science CenterErnstbrunn, Austria; ^3^Department of Cognitive Biology, University of ViennaVienna, Austria; ^4^Research Centre for Natural Sciences, Institute of Cognitive Neuroscience and Psychology, Hungarian Academy of SciencesBudapest, Hungary; ^5^Department of Medical Chemistry, Molecular Biology and Pathobiochemistry, Semmelweis UniversityBudapest, Hungary

**Keywords:** ownership style, parenting, personality, domestic dog, attachment, social support, stress coping

## Abstract

The bond dogs develop with their owner received increased attention in the last years but no study aimed at characterizing the way in which owners interact with their dogs in their daily life and how this might influence dog behavior. In order to examine how dog owners interact with their dogs, we first analyzed the behavior of 220 dog owners in 8 different standardized situations involving the owner-dog dyad. We extracted 3 behavioral factors related to “*Owner Warmth*,” “*Owner Social Support*,” and “*Owner Control*.” Further, we investigated whether owner personality, gender and age are associated with these three factors. Results indicated that older owners scored lower in “*Owner Warmth*” and in “*Owner Social Support*” and higher in “*Owner Control*” than younger owners. Furthermore, owners scoring high in “*Owner Control*” scored lower in the personality trait Openness and owners scoring high in “*Owner Social Support*” scored lower in the personality trait Conscientiousness. Finally, we also analyzed whether the dogs' reaction to an unfamiliar woman's threatening approach was associated with the owners' interaction styles. Results showed that dogs that searched for proximity of their owners during the threatening situation had owners scoring higher in “*Owner Warmth*,” as compared to dogs that reacted more autonomously, approaching the unfamiliar experimenter. Analogies between dog-owner interaction styles and human parenting styles are discussed considering the implications of the present findings for human social psychology as well as the practical relevance for dog welfare and human safety.

## Introduction

Human parenting quality strongly influences the development of children's emotional expressions, sociability, and how they cope in stressful situations (Barrett et al., 2005; Scott, 2012). Ainsworth et al. (1978) described the infant-mother relationship as attachment to the caregiver. The kind of attachment children develop to their parents has been related to different parenting styles, described by dimensions like autonomy support (Skinner et al., 2005), parental sensitivity (Belsky et al., 1991) and parental control (Barber and Harmon, 2002; Kuppens et al., 2013). More recently, researchers have suggested that within the parental sensitivity component, responsiveness to child distress and warmth in positive situations (e.g., learning and play) should be further differentiated (Davidov and Grusec, 2006). According to Belsky's (1984) theoretical work, although parenting is affected by the individual characteristics of both parents and their children, as well as the social environment they live in, the parent's individual characteristics remain the most influential factor. Supporting this conclusion, various experimental studies have linked the parents' gender, age or different personality dimensions to different parenting styles (e.g., Kochanska et al., 2004; Prinzie et al., 2009; Huver et al., 2010; de Haan et al., 2012).

Dogs have the longest history of domestication, living with humans for more than 15.000 years (Thalmann et al., 2013), and they are the most common pet animals in western households (with 14–40% of western households owning a dog, Miklósi, 2007). Dogs are mainly kept as companion animals (Bennett and Rohlf, 2007), and are often considered to be family members (Albert and Bulcroft, 1987). It has been hypothesized that pets developed specific behavioral modifications during their evolutionary history that elicit parental care in their owners (Askew, 1996; Archer, 1997; Nagasawa et al., 2015). Indeed, dogs show various infant-like behaviors, like looking at their owners and seeking proximity with them in stressful situations (Merola et al., 2012a; Gácsi et al., 2013) or showing distress signals when separated from them (Topál et al., 1998; Prato-Previde et al., 2003). Importantly, such behaviors occur specifically toward the owner, reflecting the individual bond the dog has developed to its caretaker (Merola et al., 2012b). For example, Kerepesi et al. (2015) found that most of the dogs approached by a threatening stranger ran toward their owner and not toward a familiar woman. Based on these results, it has been argued that this relationship is analogous to the attachment human infants develop to their mother during early ontogeny, and that it reflects a special and evolutionary novel capacity of domestic dogs (Miklósi and Topál, 2013).

Importantly, owners also might feel attached to their dog, and consider it as “their own child” (Rajecki and Lee Rasmussen, 1995). At a behavioral level, humans tend to address and handle dogs and children in a similar way (Mitchell, 2001; Prato-Previde et al., 2006; German, 2015). Therefore, based on the attitudes of dog owners it can be assumed that the caretaking behavior of dog owners is similar to human parenting behavior. Understanding which features are shared by human parenting and pet interaction styles can broaden our knowledge on the mechanisms underlying human social relationships and human-animal bonds, and has also practical relevance, since the owner-dog bond seems to influence a variety of dog behaviors like aggressiveness (Jagoe and Serpell, 1996) or separation-related disorders (Kobelt et al., 2003), both being an important public health concern (Casey et al., 2014).

The link between owner interaction styles and the behavior of their dogs has been investigated only partially. Kobelt et al. (2003) reported negative correlations between the amount of time the owners spend with their dogs and behavioral problems like digging, running around, chewing and escaping. Different dog-training techniques, ranging from physical punishment to reward-based training have also been compared (Hiby et al., 2004). The first one has been associated with a higher incidence of behavioral problems (e.g., aggressiveness) and lower obedience (Herron et al., 2009; Arhant et al., 2010) as well as with a smaller frequency of social interactions with an unfamiliar experimenter (Rooney and Cowan, 2011). Since positive punishment based techniques have been associated with an increase in anxiety and stress in dogs, such training has been hypothesized to weaken the dog-owner bond (Kwan and Bain, 2013). Also some other, less conspicuous elements of owner behavior have been related to dog behavior, including inconsistency and little time spent in play activities (both related to lower obedience in small dogs, Arhant et al., 2010), involvement and patience during play (positively correlated with the performance of dogs in a learning task, Rooney and Cowan, 2011), feeding the dog systematically after the owner has eaten (positively correlated with territorial aggression, Jagoe and Serpell, 1996), frequency of commands in a simple obedience task (associated with a latency of the dog obeying, Kis et al., 2012), and frequency of praising the dog (connected to an increased time spent in looking at an unfamiliar experimenter in a social learning task, Kis et al., 2012).

To date relatively few studies analyzed objectively the behavior of owners when interacting with their dog (e.g., Mitchell, 2001; Jones and Josephs, 2006; Prato-Previde et al., 2006; Horváth et al., 2008; Rooney and Cowan, 2011; Kis et al., 2012), but even in these studies the owners' behavior was recorded and linked to the behavioral components of the dogs in the same test situation (Prato-Previde et al., 2006; Horváth et al., 2008; Rooney and Cowan, 2011; Kis et al., 2012). The drawback of this is that any relationship found between owner and dog behavior could be explained by a momentary mutual influence of the two interacting individuals. A further limitation of former studies is that they assessed owner behavior in few contexts (two for, Kis et al., 2012; three for, Rooney and Cowan, 2011; one for, Horváth et al., 2008; one for, Jones and Josephs, 2006; two for, Prato-Previde et al., 2006) and mainly in positive situations (e.g., greeting, play, or learning task).

The present study had three aims: the first was to behaviorally describe the interaction style of adult dog owners in several different experimental contexts both including positive (e.g., play) and negative situations (e.g., physical restriction of the dog). Secondly, we explored whether there are associations between individual characteristics of the owners (i.e., personality, age and gender) and their dog interaction styles. Third, we investigated whether the owners' different interaction styles are associated with the reactions pet dogs showed in a socially stressful situation, namely a broadly used experimental test called Threatening approach (Vas et al., 2005; Gácsi et al., 2013). In this task, an unfamiliar person approaches the dog in a threatening manner, while the owner stands passively behind the dog. This test has been shown to activate the dogs' attachment to their owner who seem to have a safe haven effect on their dogs (Gácsi et al., 2013). Hypothesizing that owner behavior relies on mechanisms that originally evolved to promote parenting, we predicted that, similarly to parenting behavior, we would find various components of owner behavior related to support, warmth and control. We also expected that other characteristics of the owner (namely personality, gender, and age) would influence the owners' interaction styles in a similar way as they influence parenting behavior (Jones et al., 1980; Metsäpelto and Pulkkinen, 2003; Kendler and Baker, 2007; Prinzie et al., 2009; Huver et al., 2010). Finally, we expected similar associations between the owners' interaction style and their dogs' reaction to the threatening stranger. Particularly, (1) we expected a positive association between the owners' supportiveness and warmth and the dogs' proximity seeking with the owner, since a supportive and warm parenting style has been associated with more secure attachment in children; (2) we expected a positive association between the owners' warmth and the dogs' willingness to approach the experimenter in a friendly manner, based on the facilitating effect of warm parenting on the children's positive interactions with peers; and (3) we expected a positive association between the owners' level of control and higher aggression in the dogs, as a possible analogy of the effect of an authoritarian and harsh parenting on relational aggression in children (Kawabata et al., 2011).

## Methods

### Ethics statement

The owners participated in this study on a voluntary basis and the owners signed an informed consent form before the onset of the experiment. Every test was no longer than 1 h and half with a short break in the middle, and the procedure was completely non-invasive. No special permission for use of pet dogs in such socio-cognitive studies is required in Austria (Tierversuchsgesetz 2012–TVG 2012) and the experimental procedures were approved in accordance with GPS guidelines and national legislation by the Ethical Committee for the use of animals in experiments at the University of Veterinary Medicine Vienna (Ref: 09/10/97/2012 and 10/10/97/2012).

### Participants

A total of 220 owner-dog dyads participated in the present study. The test was conducted at the Clever Dog Lab, Vienna between September 2010 and November 2013. The owners (187*F*/33*M*; mean age ± *SD* = 38.64 ± 13.57 years, range 13–72 years) were recruited from the database of volunteer participants of the Clever Dog Lab. All dogs were pure-bred Border Collies [125 females (45 neutered) and 95 males (32 neutered); mean age ± *SD* = 48.07 ± 42.43 months] and were kept as family pets. We used a single breed to minimize the genetic variability of the sample, since we plan to use the same data to search for behavioral associations of genetic and epigenetic markers.

### Questionnaires

The owners provided information about their gender and age, and about the breed, sex, age, and neutered status of their dog. They were also asked to fill in the German version of the NEO-FFI personality questionnaire (Costa and McCrae, 1992; Borkenau and Ostendorf, 1993). The questionnaire includes 60 items, and it has been designed to describe the personality of the participant according to 5 dimensions: Extraversion, Neuroticism, Conscientiousness, Openness, and Agreeableness. Answers were rated on a 7-point Likert scale (ranging from 1 = I completely disagree to 7 = I completely agree).

### Procedure

Each dog-owner pair was tested in a battery of tests: 8 of these standardized situations were used to analyze the owners' behavior and, in a ninth test, we evaluated the reaction of the dogs to a human stranger approaching them in a threatening manner. The order in which the tests were performed was the same for all subjects. See Table 1 for details about the tests analyzed in this study.

**Table 1:** **Tests included in the owner interaction style analysis and the variables coded in each**.

**Name of the test**	**Type of variable**	**Variables coded**	**Descriptions**	**Data processing**	**% of individuals falling in each category (%)**
Food choice	4-points scale	Communication style	1: The O expresses their preference in a cold way and never looks at the dog; 2: The O expresses their preference in a cold way but looks at the dog at least once but no longer than for 2 seconds; 3: The O communicates with the dog using a friendly, high-pitched tone of voice and looks at the dog more than once. The O does not smile; 4: The O communicates with the dog in a friendly, high-pitched tone of voice, smiles and looks at the dog for almost the entire trial	The 6 values from the 6 trials were averaged and then rounded to full values (1, 2, 3, or 4)	1: 11.0 2: 24.8 3: 29.3 4: 34.9
DNA sample	Frequency	N° of commands	Verbal utterances pronounced using an imperative tone of voice (e.g., German equivalents of “sit!” or “stay!”)	*N* = 0 → score 1	1: 34.6
				*N* = 1–2 → score 2	2: 37.1
				*N* = 3–5 → score 3	3: 17.1
				*N* = 6–19 → score 4	4: 11.2
	Frequency	N° of attention sounds	Claps, whistles, tongue, or palatal clicks	*N* = 0 → score 1	1: 77.8
				*N* = 1–5 → score 2	2: 22.2
	Frequency	N° of petting	Pats, strokes, and scratches	*N* = 0 → score 1	1: 25.4
				*N* = 1–2 → score 2	2: 26.3
				*N* = 3–5 → score 3	3: 26.8
				*N* = 6–20 → score 4	4: 21.5
	Frequency	N° of verbal praising	Verbal utterances pronounced in a positive and friendly tone of voice (e.g., German equivalents of “Well done!”, “Super!”)	*N* = 0–4 → score 1	1: 25.4
				*N* = 5–9 → score 2	2: 26.3
				*N* = 10–14 → score 3	3: 26.8
				*N* = 15–20 → score 4	4: 21.5
	4-points scale	Active social support	1: The O restricts the movements of the dog using strength, never reassures the dog nor verbally, nor physically and speaks with the dog using a harsh tone of voice; 2: The O restricts the movements of the dog using strength, never reassures the dog nor verbally, nor physically but does not use a harsh tone of voice. 3: The O might reassure the dog verbally and/or physically but not continuously. The O speaks to the dog in gentle way and could praise the dog at the end of the test; 4: The O reassures the dog verbally and/or physically continuously. The O speaks to the dog in gentle way and praises the dog during and at the end of the test	none	1: 6.5 2: 22.1 3: 35.5 4: 35.9
Reunion after separation	4-points scale	Warmth	1: The O is avoidant and pushes down the dog if she tries to jump on her/him. The O does not greet actively the dog and could give some commands to control the behavior of the dog; 2: The O is avoidant but can accept passively the greetings of the dog. The O does not greet actively the dog and could give some commands like “sit” or “down” to control the behavior of the dog; 3: The O actively greets the dog and speaks to the dog in a friendly and high pitched tone of voice; 4: The O clearly smiles and greets the dog in an excited way speaking to the dog in a friendly and high pitched tone of voice	none	1: 2.9 2: 17.6 3: 38.5 4: 41.0
Tug-of-war play	Frequency	N° of commands	See above “DNA sample”	*N* = 0 → score 1	1: 38.0
				*N* = 1–2 → score 2	2: 24.5
				*N* = 3–5 → score 3	3: 27.4
				*N* = 6–13 → score 4	4: 10.1
	Frequency	N° of attention sounds	See above “DNA sample”	*N* = 0 → score 1	Yes: 68.6
				*N* = 1–13 → score 2	No: 31.4
	Frequency	N° of verbal praising	See above “DNA sample”	*N* = 0 → score 1	1: 24.8
				*N* = 1–2 → score 2	2: 27.6
				*N* = 3–5 → score 3	3: 20.0
				*N* = 6–20 → score 4	4: 27.6
	4-points scale	Play style	1: The O does not laugh or smile during the play session, continuously gives commands and uses a strong/harsh tone of voice. The O never allows the dog to win the game; 2: The O does not laugh or smile during the play session and might give commands to the dog using a strong/harsh tone of voice. The O never allows the dog to win the game; 3: The O is cheerful and enthusiastic during the play session but does not allow the dog to win the game; 4: The O is cheerful and enthusiastic during the play session and lets the dog win the game.	none	1: 24.2 2: 12.3 3: 43.4 4: 20.1
	3-points scale	Enthusiasm	1: The O plays with the dog showing low energy and no involvement; 2: The O plays with the dog showing medium energy and scarce involvement, 3: The O plays with the dog showing high energy and high involvement	none	1: 22.8 2: 59.8 3: 17.4
T-shirt	4-points scale	Social support	See above “DNA sample”	none	1: 7.31 2: 13.7 3: 48.9 4: 30.1
Basic commands	Frequency	N° of commands	See above “DNA sample”	*N* = 3–6 → score 1	1: 29.2
				*N* = 7–9 → score 2	2: 26.1
				*N* = 10–14 → score 3	3: 22.8
				*N* = 15–50 → score 4	4: 21.9
	Frequency	N° of petting	See above “DNA sample”	*N* = 0 → score 1	1: 13.2
				*N* = 1–2 → score 2	2: 47.9
				*N* = 3–4 → score 3	3: 19.6
				*N* = 5–10 → score 4	4: 19.3
	Frequency	N° of verbal praising	See above “DNA sample”	*N* = 0 → score 1	1: 23.2
				*N* = 1 → score 2	2: 33.3
				*N* = 2 → score 3	3: 19.8
				*N* = 3–10 → score 4	4: 23.7
	3-points scale	Authoritarian behaviors	1: The O does not raise the tone of voice neither forces the dog in a determined position; 2: The O raises the tone of the voice; 3: The O goes physically forces the dog in a determined position	none	1: 56.9 2: 12.8 3: 30.3
Teaching	4-points scale	Communication style	See above “Food choice”	none	1: 4.6 2: 21.9 3: 37.9 4: 35.6
Ball play	Frequency	N° of commands	See above “DNA sample”	*N* = 0 → score 1	1: 23.7
				*N* = 1–2 → score 2	2: 19.8
				*N* = 3–5 → score 3	3: 30.0
				*N* = 6–77 → score 4	4: 26.5
	Frequency	N° of attention sounds	See above “DNA sample”	*N* = 0 → score 1	1: 42.5
				*N* = 1–16 → score 2	2: 57.5
	Frequency	N° of verbal praising	See above “DNA sample”	*N* = 0 → score 1	1: 35.3
				*N* = 1–2 → score 2	2: 29.0
				*N* = 3–4 → score 3	3: 19.8
				*N* = 5–10 → score 4	4: 15.9
	4-points scale	Play style	See above “Tug-of-war game”	none	1: 29.7 2: 25.1 3: 36.5 4: 8.7
	3-points scale	Enthusiasm	See above “Tug-of-war game”	none	1: 13.2 2: 47.9 3: 38.9

The tests were conducted in an experimental room of the Clever Dog Lab (5 × 6 m). Before starting, the owner was informed about the procedure and was told that the aim of the study was to assess the behavior of the dog in different situations. The behavior of the dogs, their owners (O), and the experimenter (E) was continuously recorded with four cameras fixed in four corners of the room. The 4 cameras were connected to a video station outside the room. The videos were recorded and then analyzed off-line.

At the beginning of the experimental procedure the O and the dog entered the experimental room, the dog was released from the leash and was free to explore the room for 2 min. The E gave instructions to the O before each test.

#### Owner interaction style tests

The following tests and variables were used to assess the owners' behavior (see Table 1 for a detailed description of all variables coded):

##### Food choice

The O was asked to show a clear preference for an empty plate over another that had some food on in order to see whether O can get his/her dog to make a counterproductive choice. First, the E positioned 2 plates on the floor, one with a piece of food on it and another one empty. The O was sitting on a chair on one side of the room and the dog was attached on a leash on the other side. When the plates were in place and E went behind the dog, O got up from her/his chair, crouched close to the empty plate, picked it up and acted as if it would be really interesting and delicious for the dog. Then O returned to his/her chair and the dog was released to make a choice. The procedure was repeated 6 times. In each trial we scored the warmth and the enthusiasm of O's communication on a 4-points scale taking into account the following behaviors: looking into the eyes of the dog, smiling, speaking with a high-pitched, friendly tone of voice.

##### DNA sample collection

The O was informed that the E would take a DNA sample from the inner side of the dog's mouth by gently rotating a cotton swab, a procedure that caused no pain to the dog but could still be potentially slightly stressful to them because of the mild restrain and the closeness of the unfamiliar E. O was asked to help the E to take the sample by holding the dog by the collar/harness and by trying to keep it calm. We counted the number of commands, attention sounds (e.g., claps and whistles), petting (number of gentle touches) and verbal praising (number of utterances) the O used. We also scored the active social support O provided to the dog on a 4-points scale taking into account the following behaviors: speaking to the dog, physical strength used to hold the dog, friendly tone of voice.

##### Reunion after separation

We coded how the O greeted his/her dog after separation. The O reentered the room after 2 min the dog spent without her/him in the room. The O was instructed to ignore the dog for the first 5 s but then to interact with the dog like he/she wanted. We scored the warmth and the enthusiasm of O's greeting on a 4-points scale taking into account the following behaviors: looking into the eyes of the dog, smiling, speaking with a high-pitched, friendly tone of voice, avoidance.

##### Tug-of-war play

The O was asked to play a tug-of-war game with the dog using a rope provided by the E for 30 s. No further instructions were given, the dyad could play as they usually do. We coded the number of verbal orders the O issued during the play session, the number of non-verbal sound signals used by the O to attract the dogs' attention (e.g., whistle, clapping) and the number of praises. We also scored the play style showed by the O during play on a 4-points scale taking into account the following behaviors: looking into the eyes of the dog, smiling, speaking with a high-pitched, friendly tone of voice, letting the dog win the game. Furthermore, we scored the energy and enthusiasm showed by the O while playing with the dog on a 3-points scale.

##### T-shirt

The E asked O to put a T-shirt on the dog. This was a task that was novel to our subjects (none of the dogs had worn a human T-shirt before) and might have again be somewhat challenging because it required physical manipulation of the dog's body and because the O was not allowed to say anything to the dog during the test. We also scored the active social support O provided to the dog on a 4-point scale taking into account the following behaviors: looking at the dog, smiling, physical strength used to manipulate the dog.

##### Basic commands

The O commanded the dog to sit and lay down and to stay in a specific area of the room, while the E was kneeling at the opposite side of the room, and tried to attract the dog's attention by fumbling in a plastic box containing crumpled newspapers that made rustling noises. If the O succeeded in having the dog stay in that position, he/she was asked to join the E and to call the dog to him/her after 15 s. The O was not allowed to offer any reward to the dog during the test. We coded the number of verbal commands that O gave to the dog, the number of praises, the number of petting and whether the O showed authoritarian behaviors during the task like raising the tone of voice and/or physically forcing the dog on a determined position.

##### Teaching

The O had to show the dog how to reach a reward in a bin covered by a lid. The O was instructed to crouch next to the bin while the dog was attached on a leash to the wall, facing the O. The O was asked then to call the name of the dog, show them a piece of food, remove the lid of the bin, put the food in the bin and finally put the lid back on the bin. This procedure was repeated 4 times, after which the dog was allowed to manipulate the bin in order to get access to the reward. We scored the warmth and the enthusiasm of O's communication on a 4-points scale taking into account the following behaviors: looking into the eyes of the dog, smiling, speaking with a high-pitched, friendly tone of voice.

##### Ball play

The O was asked to play a retrieval game with their dog using a ball. The O was instructed to throw the ball to the other side of the room 3 times and to ask the dog to bring it back to them. We coded the number of verbal orders the O issued during the play session, the number of non-verbal sound signals used by the O to attract the dogs' attention, (e.g., whistle, clapping) and the number of praises. We also scored the play style showed by the O during play on a 4-points scale taking into account the following behaviors: looking into the eyes of the dog, smiling, speaking with a high-pitched, friendly tone of voice, letting the dog win the game. Furthermore, we scored the energy and enthusiasm showed by the O while playing with the dog on a 3-points scale.

All videos were coded by the same coder (GC) and 20% of the videos were independently analyzed by a second coder who was naive to the hypotheses of the present study. Agreement between the 2 coders was good or excellent according to the variable (Cohen's κ ranging between 0.72 and 0.94).

#### Threatening approach test

In order to assess in each dyad how the dogs would seek for support from their owners in case of a social threat, we used a similar procedure as described by Vas et al. (2005). The dog, held on leash by O, was approached by E in a threatening manner: starting from a position 5 m away from the dog, E walked very slowly toward the dog (one step ca. every 4 s) while bending her upper body slightly and looking steadily in the eyes of the dog. The test was no longer than 60 s and was terminated when the E reached the dog or when the dog approached the E or when the dog withdrew behind the O or when the dog was openly aggressive (continuously growling and snapping at the E). After the test E crouched down and comforted the dog by petting and/or talking gently to them. During the entire procedure, the O stood behind the dog silently and without moving, and did not interact with his/her dog in any way. We coded whether at any point throughout the E's approach (till the E made the last step toward the dog) the dog made an attempt to approach the E in a friendly (0.1), appeasing (0.1) or aggressive manner (0.1), whether they remained passive (0.1) or they hid behind the O (0.1) while the E was walking toward them. Furthermore, we coded the final reaction of the dog shown when the E made the last step toward them. We named this variable “Reaction at the end of the test” and we coded in an exclusive manner whether it was Aggressive, Friendly/Appeasing, Passive or a Retreat (see Table 2 for variables definitions). In regard to score “Friendly/Appeasing Reaction at the end of the test,” initially we coded friendly and appeasing approaches separately but out of the 66 dogs that received this score only 5 dogs approached the E in a friendly way without showing any sign of submission. Therefore, we decided to group all dogs that showed a friendly or appeasing behavior as a final reaction together.

**Table 2:** **Behavior variables scored during the threatening approach test**.

**Variable**	**Type of variable**	**Definition of the variable**	**% of individuals falling in each category (%)**
Friendly	0/1	The dog steps toward the E visibly wagging its tail	0: 93.5
			1: 6.5
Appeasing	0/1	The dog steps toward the E with tail between its legs, its ears pulled back and with a tense body posture	0: 53
			1: 47
Aggression	0/1	The dog is showing at least one of the following behaviors: growling, snarling, snapping	0: 85.4
			1: 14.6
Hiding behind the owner	0/1	The dog withdraws so that the owner is positioned between itself and the E	0: 79.6
			1: 20.4
Passive	0/1	The dog remains standing or sitting without showing a specific reaction	0: 86.6
			1: 13.4
Reaction at the end of the test	score	Aggressive: the dog growls, snarls or snaps at the E;	Aggressive: 33.6
		Friendly/Appeasing: the dog moves toward the E wagging its tail or with tail between its legs, its ears pulled back and with a tense body posture;	Friendly/Appeasing: 31.3
		Passive: the dog remains passive, shows no visible reaction;	Passive: 10.4
		Retreat: the dog retreats making more than one step in direction of the O	Retreat: 24.7

A proportion of videos of the Threatening approach test (20%) was double coded by 2 independent coders and agreement between the 2 was moderately good or very good (Cohen's κ ranging between 0.54 and 0.89). The Owner Interaction Style tests and the Threatening Approach test were coded by different coders which were blind to the outcomes of the coding of the other test. The anonymized dataset has been made available and added as Supplementary Material.

### Statistical analysis

To describe the owner interaction styles, we applied an Exploratory Factor Analysis (EFA) with Oblimin rotation on the behavioral variables recorded in the Owner interaction style test. The internal consistency of the extracted factors was investigated by calculating Cronbach's alpha. To investigate associations between the owners' individual characteristics and their interaction styles, we ran General Linear Models (GLM) with the Owner interaction style factors extracted from the EFA as dependent variables and the owners' age, gender and five personality factors obtained from the NEO-FFI questionnaires as predictors. Non-significant predictors (*p* > 0.05) were removed from the model and are not reported in the results. Model residuals were tested for normality using the Shapiro-Wilk normality test and homoscedasticity was assessed via plots of residuals against fitted values. Finally, to investigate associations between the owner interaction styles and dog behavior during the Threatening approach test, we ran a Multinomial Regression model with the “Reaction at the end of the test” as a dependent variable and the age, sex, neutered status of the dog, the interaction between the neutered status of the dog and their sex, and the owner interaction style factors as independent variables. We then ran Generalized Linear Models (GZLM) with binomial distribution to check whether dogs' behavior showed throughout the test were associated with different owner interaction styles. We ran five models with the following variables as response variables: “Aggression,” “Appeasing,” “Friendly,” “Hide behind the owner,” and “Passive.” The predictors were the same as for the Multinomial Regression model. We selected the best model using model reduction based on *p*-values. We accounted for multiple testing using post-hoc sequential Bonferroni (Holm, 1979). In addition, for exploratory purposes, we present the effect sizes of the associations between the variables included in the present study in **Tables 4, 5**. All statistical tests were conducted using the software R (version: 3.1.1, R Core Team, 2014), except of the EFA for which we used IBM SPSS Statistics v. 24. All *p*-values are two-tailed and the level of significance was set to *p* < 0.05.

## Results

### Components of owner interaction style

Based on the Scree plot, 3 components were extracted from the EFA which account for 29.47% of the total variance (KMO = 0.69, see Table 3) and included a total of 20 variables (out of 23). The first component, labeled as “*Owner Warmth*”, was composed of 9 variables measured in 5 sub-tests, mostly related to the warmth, communication style and enthusiasm showed by the owner in positive situations (i.e., food choice, reunion after separation, tug-of-war play, ball play and teaching). The second component, labeled as “*Owner Social Support*,” was composed of 6 variables measured in 3 sub-tests, mostly related to petting and praising that the owner provided to the dog in stressful situations (i.e., DNA sample collection, T-shirt test and Basic commands). The third component, labeled as “*Owner Control*,” was composed of 5 variables measured in 3 sub-tests, mostly related to the number of commands that the owner gave to the dog either during play situations (ball play and tug-of-war play) or during the basic commands task. The internal consistency (Cronbach's alpha) of the “*Owner Warmth*” and “*Owner Social Support*” factors was adequately high (“*Owner Warmth*:” α = 0.77; “*Owner Social Support*:” α = 0.68), however the consistency of the factor called “*Owner Control*” was lower (α = 0.49).

**Table 3:** **Rotated factor matrix restricted to three factors**.

	**Factors**		
**Variable (sub-test)**	**Warmth**	**Social support**	**Control**
Enthusiasm (Ball play)	**0.76**	0.34	−0.08
Enthusiasm (Tug-of-war play)	**0.67**	0.21	0.18
Praising (Ball play)	**0.52**	0.24	−0.04
Praising (Tug-of-war play)	**0.36**	0.15	0.19
Play style (Ball play)	**0.51**	0.21	−0.48
Play style (Tug-of-war play)	**0.57**	0.32	−0.12
Warmth (Reunion after separation)	**0.52**	0.30	−0.12
Communication style (Teaching)	**0.48**	0.31	−0.05
Communication style (Food choice)	**0.42**	0.19	0.08
Social support (DNA sample)	0.37	**0.75**	−0.04
Social support (T-shirt)	0.26	**0.37**	−0.16
Petting (DNA sample)	0.20	**0.62**	−0.08
Petting (Basic obedience)	0.14	**0.40**	−0.03
Praising (DNA sample)	0.29	**0.49**	0.19
Praising (Basic obedience)	0.22	**0.49**	0.04
Commands (Ball play)	0.03	0.04	**0.49**
Commands (Tug-of-war play)	−0.06	−0.07	**0.33**
Attention sounds (Tug-of-war play)	0.22	0.10	**0.36**
Commands (Basic obedience)	0.03	0.01	**0.38**
Eigenvalue	4.11	2.06	1.72
Variance explained	17.41	6.64	5.41
Cronbach's alpha	0.77	0.68	0.49

*Behaviors loadings in each factor in boldface*.

### Do owners' age, gender and/or personality predict their interaction style?

**Figure 1:**
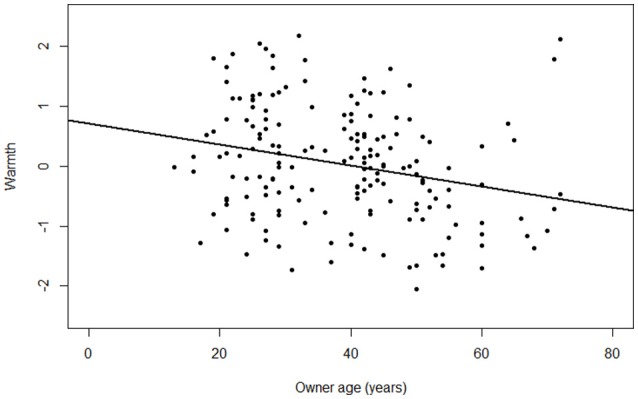
**Relationship between the factor “*Owner Warmth*” and the owner age (expressed in years)**.

In the present study, Cronbach's alpha for each personality dimension were: Extraversion (α = 0.75), Neuroticism (α = 0.85), Conscientiousness (α = 0.81), Openness (α = 0.74) and Agreeableness (α = 0.76). We found that younger owners scored higher on “*Owner Warmth*” than older owners [estimate ± *SE* = −0.02 ± 0.005, *t*_*(146)*_ = −3.17, *p* < 0.01; Figure 1] and that owners' Openness was positively associated with “*Owner Warmth*” [estimate ± *SE* = 0.35 ± 0.16, *t*_*(146)*_ = 2.20, *p* = 0.03]. Furthermore, we found that younger owners scored higher in “*Owner Social Support*” than older owners [estimate ± *SE* = −0.02 ± 0.005, *t*_*(146)*_ = −3.56, *p* < 0.01] and that owners' Conscientiousness was negatively associated with “*Owner Social Support*” (estimate ± *SE* = −0.30 ± 0.14, *t*_*(146)*_ = −2.15, *p* = 0.03). In addition, we found that owners' Openness was negatively associated with “*Owner Control*” [estimate ± *SE* = −0.38 ± 0.13, *t*_*(156)*_ = −2.81, *p* < 0.01]. Correlations between all variables included in this section are reported in Table 4.

**Table 4:** **Correlations between Owner interaction styles, owner demographic characteristics and owner personality factors (Pearson's *r* and descriptive statistics)**.

	**Owner gender**	**Owner age**	**Neuroticism**	**Extraversion**	**Openness**	**Agreeableness**	**Conscientiousness**	**Owner warmth**	**Owner control**		
	**Pearson's *r***									***M***	***SD***
Owner gender	−										
Owner age	0.14^*^	−								38.64	13.57
Neuroticisms	0.21^**^	−0.16^*^	−							1.54	0.68
Extraversion	0.15^*^	−0.17^*^	−0.34^**^	−						2.52	0.46
Openness	0.10	0.09	−0.02	0.02	−					2.57	0.48
Agreeableness	0.20^**^	0.06	−0.12	0.26^**^	0.08	−				2.72	0.43
Conscientiousness	0.07	0.11	−0.33^**^	0.26^**^	−0.13	−0.00	−			2.92	0.48
Owner warmth	0.15^*^	−0.25^**^	0.11	0.04	0.12	−0.03	−0.09	−		0.04	0.93
Owner control	0.04	0.12	−0.00	−0.04	−0.22^**^	0.04	0.02	−0.03	−	0.02	0.81
Owner social support	0.13	−0.24^**^	0.01	0.04	0.04	−0.07	−0.16^*^	0.53^**^	−0.05	0.02	0.86

* *p < 0.05*;

** *p < 0.01. Significant correlations (p < 0.05) are presented boldfaced*.

### Factors influencing dog behavior

**Figure 2:**
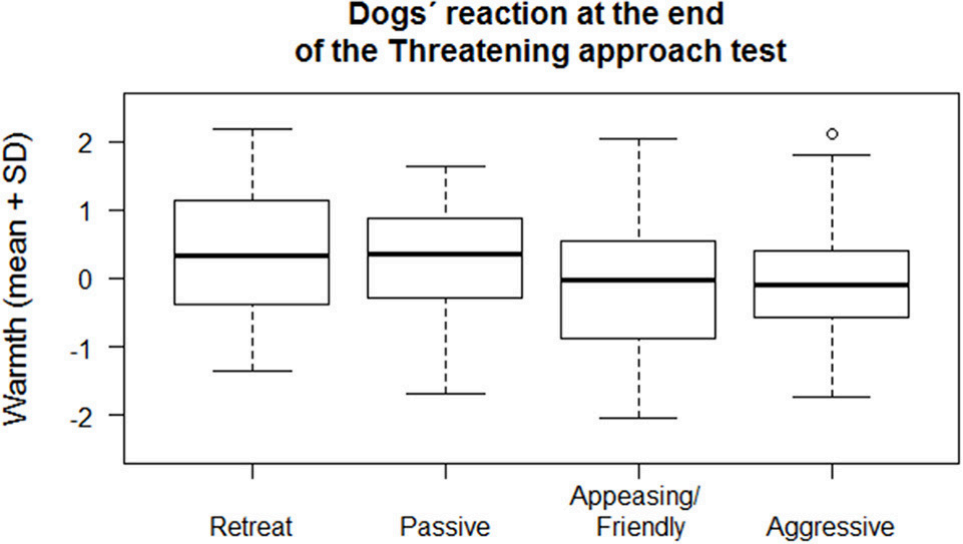
**Mean and standard deviation (*SD*) of the factor called “*Owner Warmth*” in relation to the reaction dogs showed at the end of the Threatening approach test (Aggressive, Friendly/Appeasing, Passive, Retreat)**.

**Figure 3:**
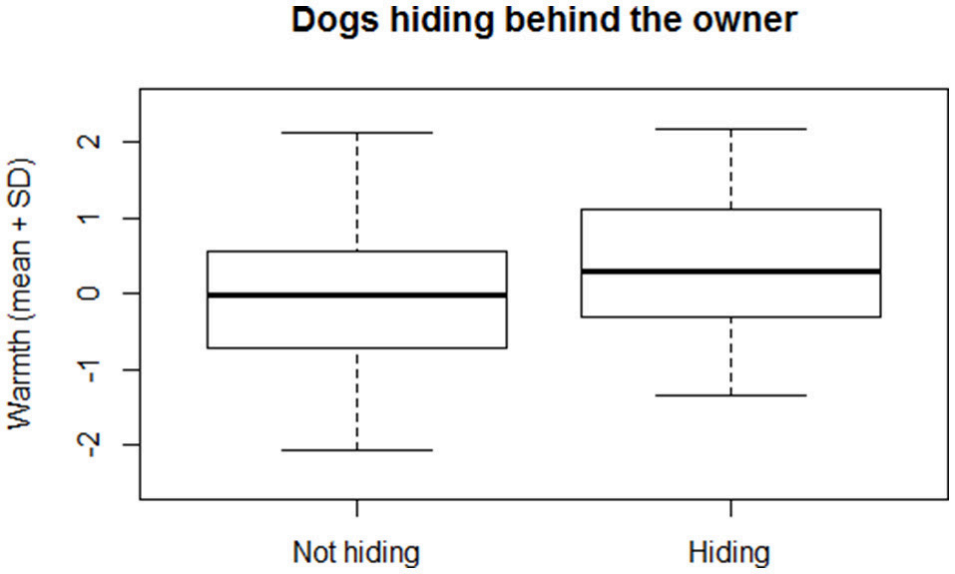
**Mean and standard deviation (*SD*) of the factor “O*wner Warmth*” in dogs who hid or did not hide behind the owner during the Threatening approach test**.

We found that dogs that approached the E either in a friendly/appeasing or in an aggressive manner at the end of the Threatening approach test (variable “Reaction at the end of the test”) had owners scoring lower in the “*Owner Warmth*” factor, while those dogs who remained passive or that hid behind the O had owners scoring higher in the “*Owner Warmth*” factor (Multinomial Regression model, χ^2^ = 8.94, *df* = 3, *p* = 0.03; Figure 2). On the other hand, during the test, we found that those dogs that showed signs of aggression toward the E, were more likely to be intact [estimate ± *SE* = −1.27 ± 0.058, *z*_*(172)*_ = −2.20, *p* = 0.03] and that they had a tendency to have owners scoring higher in “*Owner control*” [estimate ± *SE* = −0.49 ± 0.27, *z*_*(171)*_ = 1.83, *p* = 0.07]. Interestingly, there was no difference between neutered or intact males and females, since the interaction neutered status*sex of the dog was not significant [estimate ± *SE* = 0.88 ± 1.05, *z*_*(194)*_ = −0.84, *p* = 0.40]. Furthermore, those dogs that approached the E in an appeasing way had owners scoring lower in the “*Owner Warmth*” [estimate ± *SE* = 0.29 ± 0.16, *z*_*(185)*_ = −1.78, *p* = 0.07] and those dogs that approached the E in a friendly way tended to be younger than those who showed no friendly behavior [estimate ± *SE* = −0.02 ± 0.01, *z*_*(214)*_ = −1.89, *p* = 0.06]. Age affected also the likelihood to remain passive: in fact, older dogs were more likely to show no reaction to the E than younger dogs [estimate ± *SE* = 0.01 ± 0.004, *z*_*(214)*_ = 2.87, *p* < 0.01]. In addition, those dogs that hid behind the owner had owners scoring higher in “*Owner Warmth*” [estimate ± *SE* = 0.44 ± 0.20, *z*_*(186)*_ = 2.14, *p* = 0.03; Figure 3]. Correlations between all variables included in this section are reported in Table 5.

**Table 5:** **Correlations between Owner interaction styles, dog demographic characteristics, dog behavior during the Threatening approach test (Pearson's *r* and descriptive statistics)**.

	**Dog sex**	**Neutered status**	**Aggression**	**Passive**	**Appeasing**	**Friendly**	**Hiding behind the owner**	**Owner warmth**	**Owner control**	**Owner social support**		
	**Pearson's *r***										***M***	***SD***
Dog sex	−											
Neutered status	0.01	−										
Aggression	0.04	0.17^*^	−									
Passive	0.04	0.09	0.12	−								
Appeasing	0.01	0.11	0.28^**^	0.09	−							
Friendly	0.11	0.11	0.06	0.10	0.13	−						
Hiding behind the owner	0.02	0.07	0.04	0.20^**^	0.25^**^	0.09	−					
Owner warmth	0.01	0.02	0.08	0.09	0.13	0.06	0.16^*^	−			0.04	0.93
Owner control	0.17^*^	0.03	0.15^*^	0.06	0.00	0.10	0.04	−0.03	−		0.02	0.81
Owner social support	0.03	0.05	0.04	0.01	0.04	0.05	0.03	0.53^**^	−0.05	−	0.02	0.86
Dog age	0.04	0.31^**^	0.01	0.20^**^	0.04	0.14^*^	0.11	0.08	−0.07	−0.09	48.07	42.43

* *p < 0.05*;

** *p < 0.01. Significant correlations (p < 0.05) are presented boldfaced*.

## Discussion

The aim of this study was to characterize the way in which owners interact with their dogs, which individual characteristics could influence it and to determine what kind of owner behavior is associated with which dog behavior. By testing a large sample of dog-owner dyads in a number of experimental behavioral tasks, we found and described three different dimensions of owner interaction style: warmth and enthusiasm in positive contexts, social support in stressful situations and behavioral control. Moreover, we found evidence that owner age and two personality factors (Openness and Conscientiousness) significantly modulated these three distinct components of owner interaction style, and we found associations between the warmth of the owner and the behavior of the dog in a stressful social situation.

### Different dimensions of owner interaction styles

The three owner interaction dimensions that emerged in the present study are consistent with those described in human parenting. “*Owner Warmth*” (the affection, warmth and enthusiasm showed by the owner in positive situations like playing, teaching a new task, reunion with the dog after a short period of separation) can be compared to the parenting dimension labeled as “Warmth,” described as the cluster of parent behaviors resulting in positive affection, fondness and enjoyment (Gottman et al., 1996; Davidov and Grusec, 2006). On the other hand, “*Owner Social Support*” provided to the dog during stressful situations e.g., during physical restriction (DNA sample collection and T-shirt test) or in an obedience task, where distraction made the dogs' task difficult, can be related to the parenting factor called “Responsiveness to distress” (Davidov and Grusec, 2006; Gottman et al., 1996). “*Owner Control*,” the third dimension of ownership style we detected (based on the number of commands and attention calling given by the owner during play and in an obedience task) also shows some analogies with “Parental Control” (Barber and Harmon, 2002; Kuppens et al., 2013).

### Factors affecting owner behavior

Since the Big Five personality dimensions, gender and age have been associated with different parenting styles (Prinzie et al., 2009), we analyzed whether this would be the case also in regard to owner interaction styles. We did not find any effect of the owners' gender on their interaction style, but we found some evidences that older owners were less warm and provided less social support in stressful situations than younger owners. This is in line with other research showing that older people are less attached to their dogs (Netting et al., 2013), they build a less beneficial bond with them, see them less as persons they can communicate with, and establish more boundaries for their dogs (Dotson and Hyatt, 2008). Aging might alter the way owners perceive their dogs' emotional states and needs and, therefore, could make them less responsive to their dogs (Sullivan et al., 2007; Slessor et al., 2008). Another possibility is that older owners (that is, people who started to keep dogs earlier and learned dog training earlier) embrace a more old-fashioned way of interacting with their dogs, more detached, more controlling, and less supportive (Hiby et al., 2004). We found three associations between the personality of the owners and their behavior showed in our experimental tasks. In particular, the personality trait called Openness was positively associated with “*Owner Warmth*,” and negatively associated with “*Owner Control*” (but see Kis et al., 2012 for opposite results) while Conscientiousness was negatively associated with “*Owner Social support*.” Openness to experience characterizes people who are curious, imaginative, insightful, etc., and various associations between this personality trait and parenting have been reported: parents scoring high in Openness are more empathic (Kochanska et al., 2004), more open to nontraditional parenting approaches (de Haan et al., 2012), have a higher degree of parental positive control (defined as setting consistent and appropriate limits, as well as an appropriate level of response to the child's actions), provide more autonomy support (Prinzie et al., 2009) and show less negative control than those scoring lower in Openness (Karreman et al., 2008). Conscientiousness, on the other hand, characterizes people who are careful, organized and goal-oriented (Prinzie et al., 2009), and has been positively associated with parental warmth, behavioral control (Prinzie et al., 2009) and responsiveness (Clark et al., 2000). In our study, the association between Openness and “*Owner Warmth*” as well as “*Owner Control*” seems to reflect a similar contribution to how this personality dimension is linked to parental behavior, while the negative association between Conscientiousness and “*Owner Social Support*” suggests a different link between Conscientiousness and the way in which owners vs. parents interact with their dogs or children in stressful situations. This deviation might be partly explained by the owners' having been instructed how to perform specific tasks which might have prevented more conscientious owners from showing spontaneous, supportive behaviors like praising and petting the dog.

### Owners interaction styles and dog behavior

We have found evidence that the dogs' reaction to an unfamiliar person approaching them in a challenging way was associated with owners' interaction style. Dogs, whose owners showed a warmer behavior and more enthusiasm in different positive situations, relied more on their owners: they were more likely to hide behind their owners, less likely to approach the experimenter in an appeasing manner and more likely to remain closer to their owners also at the end of the test, when the experimenter was closer and therefore more threatening. This behavior of the dogs has been suggested to reflect the safe haven effect of the owners on their dogs (Gácsi et al., 2013). The safe haven effect is a central feature of the parent-infant attachment: securely attached infants seek for proximity to their caregiver in stressful and dangerous situations (Bowlby, 1969). Of the various component of human parenting, maternal sensitivity has been linked to the safe haven effect and to attachment security in general in human infants (Leerkes, 2011). Paralleling this, we found that dogs were more likely to seek for proximity to their owners when those scored higher in “*Owner Warmth*.”

We found no association between the dogs' reaction and the “*Owner Social Support*,” contrary to our predictions. We expected this factor to be associated with more proximity seeking by the dogs analogously to recent human findings suggesting that the parents' responsiveness to infant distress rather than to non-distress predicts children attachment security (Davidov and Grusec, 2006; Leerkes, 2011). A possible explanation is that our “*Owner Social Support*” factor was mainly constituted of the amount of petting and praising given by the owner in mildly stressful contexts, but we did not take into account whether these comforting behaviors were at that moment appropriate responses or not. This means that the owners who scored high on “*Owner Social Support*” may be real supportive owners (who gave an appropriate response to their dog's distress) or may be overprotective or anxious owners who praised and petted their dogs despite these were not perceiving the situation as stressful. In humans, overprotective parenting is associated with children's anxiety and difficulties in coping with stressful social situations (Spokas and Heimberg, 2009; Gere et al., 2012), leading to opposite outcomes than supportive parenting (Gottman et al., 1996). Therefore, in future studies it will be important to distinguish between these two different constructs (overprotective vs. supportive interaction style) also in dog owners, in order to assess whether and how “*Owner Social Support”* effects dog behavior.

Regarding the “*Owner Control*” factor we expected a positive association between a more controlling owner interaction style and higher aggression in dogs, as a possible analogy of the effect of an authoritarian and harsh parenting on relational aggression in children (Kawabata et al., 2011). But we found that “*Owner Control*” only tended to be associated with the dogs' likelihood to show aggressive reaction during the Threatening approach.

Beyond owner influence, we found that dogs' behavior varied also with their age, gender and neutered status. In particular, we found that intact dogs (independently from their sex) were more likely to approach the experimenter in an aggressive way. Our results confirm former studies in which intact males were reported to be more aggressive than neutered males (Gershman et al., 1994; Roll and Unshelm, 1997), and intact individuals were bolder than neutered ones (Kubinyi et al., 2009). However, other studies found that neutered dogs were more likely to bite than intact ones (Guy et al., 2001) or that neutering did not decrease the incidence of aggression (Podberscek and Serpell, 1996). In addition, we found that younger dogs tended to more likely approach the experimenter in a friendly way, while older dogs remained rather passive than younger dogs. In harmony with our findings, in a similar experimental task (Horváth et al., 2007), older dogs were more passive than younger dogs and younger dogs tended to be more pro-active than older dogs. The authors argued that older dogs may be simply physically less active or they may be less able to cope with stressful situations making them more passive in complex social interactions.

### Strengths, limitations, and future directions

In a novel way, this study has combined different methods (behavioral observations and questionnaires) and used many different contexts, both positive and negative, to assess dog owners' interaction styles quantitatively, and statistical analyses (factor analysis) to minimize the impact of coder perception. Such a methodological approach is rare in human psychological research (as also pointed out by Power, 2013 in a recent review). Still, our study led to similar dimensions described in the human parenting literature, confirming the validity of human parenting dimensions described by more subjective methods, and suggesting that these dimensions might be related to human care-giving in general. Furthermore, since owner personality correlated only partially with owner behavior, we could argue that possible differences in owner behavior are not only derived from individual characteristics, but also from socio-cultural differences, education and/or experience with dogs. These could be part of the elements characterizing the social context in which the dog-owner relationship develops (similarly to what (Belsky, 1984) defines as the social environment in which the parent-infant relationship is embedded). Future studies would be needed to address the effect of these factors on the owners' interaction style.

We tested a large sample of dog-owner dyads (at least for animal behavioral studies), and we could exclude some confounding factors like breed differences since all our dogs were pure bred Border Collies kept as pets. On the other hand, future studies need to investigate to what extent our findings can be generalized to other dog breeds and to dogs used in working settings (e.g., police or guide dogs). It also remains to be investigated how other individual characteristics of dogs, such as their personality, negative experiences with individuals other than the owner, living in the city or in rural areas with more or less contact with unfamiliar people, may affect both the behavior of the dogs and their owners' interaction style.

In this study, we provided additional evidences to the view of the dog-owner relationship as an individualized and complex system, which analysis has important welfare implications for both dogs and humans. Dogs live in a social environment shared with humans and if they reacted to potentially stressful situations with aggressive behaviors they would represent a danger not only for the humans around them but also for themselves, since aggression is a common cause of relinquishment of pet dogs (Casey et al., 2014). Owners are in the best position to prevent such situations if provided with the necessary knowledge on how to control, support and communicate with their dogs. Finally, the observations here presented could serve as a basis for the development of new practical tools to help the owners to better interact with their dogs and, ultimately, to improve the dog-owner relationship.

## Author contributions

GC and ZV designed the study; BT, FR, and ZV prepared the study material and data acquisition; GC, ZB, and BT entered the data and prepared it for statistical analyses; GC, ZB, and BT analyzed the data; GC, BT, ZV, interpreted the data; ZV and FR obtained funding; GC wrote the first draft of the manuscript; GC, BT, ZB, FR, ZV critically revised the manuscript for important intellectual content. All authors gave final approval of the manuscript version to be published and agreed to be accountable for all aspects of the work in ensuring that questions related to the accuracy or integrity of any part of the work are appropriately investigated and resolved.

## Funding

This research was supported by the Austrian Science Fund (FWF) project I 1271-B24 and P21418 and the Hungarian Scientific Research Fund project OTKA-ANN 107726. We further thank our sponsor Royal Canin for supporting the Clever Dog Lab.

### Conflict of interest statement

The authors declare that the research was conducted in the absence of any commercial or financial relationships that could be construed as a potential conflict of interest.
